# Comparative Analyses of Lung Transcriptomes in Patients with Alveolar Capillary Dysplasia with Misalignment of Pulmonary Veins and in *Foxf1* Heterozygous Knockout Mice

**DOI:** 10.1371/journal.pone.0094390

**Published:** 2014-04-10

**Authors:** Partha Sen, Avinash V. Dharmadhikari, Tadeusz Majewski, Mahmoud A. Mohammad, Tanya V. Kalin, Joanna Zabielska, Xiaomeng Ren, Molly Bray, Hannah M. Brown, Stephen Welty, Sundararajah Thevananther, Claire Langston, Przemyslaw Szafranski, Monica J. Justice, Vladimir V. Kalinichenko, Anna Gambin, John Belmont, Pawel Stankiewicz

**Affiliations:** 1 Department of Pediatrics, Baylor College of Medicine, Houston, Texas, United States of America; 2 Department of Molecular & Human Genetics, Baylor College of Medicine, Houston, Texas, United States of America; 3 Interdepartmental Program in Translational Biology and Molecular Medicine, Baylor College of Medicine, Houston, Texas, United States of America; 4 Department of Pathology, University of Texas MD Anderson Cancer Center, Houston, Texas, United States of America; 5 Division of Pulmonary Biology, Cincinnati Children's Hospital Research Foundation, Cincinnati, Ohio, United States of America; 6 Institute of Informatics, University of Warsaw, Warsaw, Poland; 7 Department of Epidemiology, University of Alabama at Birmingham, Birmingham, Alabama, United States of America; 8 Robinson Research Institute, School of Pediatrics and Reproductive Health, University of Adelaide, Adelaide, Australia; 9 Department of Pathology, Baylor College of Medicine, Houston, Texas, United States of America; 10 Mossakowski Medical Research Center, Polish Academy of Sciences, Warsaw, Poland; Emory University School of Medicine, United States of America

## Abstract

Alveolar Capillary Dysplasia with Misalignment of Pulmonary Veins (ACDMPV) is a developmental disorder of the lungs, primarily affecting their vasculature. *FOXF1* haploinsufficiency due to heterozygous genomic deletions and point mutations have been reported in most patients with ACDMPV. The majority of mice with heterozygous loss-of-function of *Foxf1* exhibit neonatal lethality with evidence of pulmonary hemorrhage in some of them. By comparing transcriptomes of human ACDMPV lungs with control lungs using expression arrays, we found that several genes and pathways involved in lung development, angiogenesis, and in pulmonary hypertension development, were deregulated. Similar transcriptional changes were found in lungs of the postnatal day 0.5 *Foxf1* ^+/−^ mice when compared to their wildtype littermate controls; 14 genes, *COL15A1, COL18A1, COL6A2, ESM1, FSCN1, GRINA, IGFBP3, IL1B, MALL, NOS3, RASL11B, MATN2, PRKCDBP,* and *SIRPA,* were found common to both ACDMPV and *Foxf1* heterozygous lungs. Our results advance knowledge toward understanding of the molecular mechanism of ACDMPV, lung development, and its vasculature pathology. These data may also be useful for understanding etiologies of other lung disorders, e.g. pulmonary hypertension, bronchopulmonary dysplasia, or cancer.

## Introduction

Alveolar Capillary Dysplasia with Misalignment of Pulmonary Veins (ACDMPV; MIM# 265380) is a rare developmental disorder of the lung [1]. ACDMPV was first described by McMahon [2] and its genetic basis emerged as more patients [3] and familial cases were reported [4]. The primary diagnostic features of ACDMPV include misalignment (malposition) of pulmonary veins, medial thickening of smooth muscles in pulmonary arteries, hyperplasia of alveolar epithelium, and drastically decreased number of capillaries and lobular underdevelopment [5]. Approximately one third of the patients also have lymphangiectasis. The disease usually presents within a few hours after birth although late presentations have been reported [6], [7]. Treatment, including high pressure oxygen, nitric oxide, extra corporeal membrane oxygenation (ECMO) [8]–[11], and more recently Sildenafil [12], only provide temporary relief as the disease is uniformly lethal. Most patients die within the first few weeks of life although some prolonged survivals have been described [13]. The majority of the patients with ACDMPV also have extra-pulmonary anomalies, including gastrointestinal, cardiovascular, and genitourinary systems [14].

We have shown that ACDMPV results from haploinsufficiency of *FOXF1* [15]. To date 42 point mutations and 18 genomic deletions involving *FOXF1* or its upstream regulatory region have been identified in patients with ACDMPV [15]–[17]. Interestingly, our recent work has indicated that *FOXF1* is incompletely paternally imprinted in human lungs [17], [18]. *Foxf1* ^+/−^ mice exhibit neonatal mortality similar to human infants with ACDMPV and die due to defects in vasculogenesis and alveolarization [19]. They also demonstrate other foregut malformations such as narrowing of the esophagus and trachea, esophageal atresia and trachea-esophageal fistula [20]. Unlike in humans, *Foxf1* was not found to be imprinted in mice (data not shown).

To better understand the molecular bases of ACDMPV and to identify major genes and pathways involved in lung development, we performed comparative analyses of lung transcriptomes in patients with ACDMPV and in *Foxf1* ^+/−^ mice using Human Ref-8 v3 and Mouse WG-6 v2 Expression BeadChip Illumina microarrays, respectively.

## Materials and Methods

### Ethics Statement

Patients with histopathologically-verified ACDMPV were recruited after signing consent for the study, Institutional Review Board (IRB) protocol # H-8712 “Molecular genetics of Alveolar capillary dysplasia with misalignment of pulmonary veins (ACDMPV)” approved by the IRB for Baylor College of Medicine (BCM) and Affiliated Hospitals. All mouse experiments were carried out under the approval of the Institutional Animal Care and Use Committee (IACUC) at BCM. Mice were housed in the Transgenic Mouse Facility (barrier level 3) under the care of the Center for Comparative Medicine (CCM), which is accredited by the Association for Assessment and Accreditation of Laboratory Animal Care (AAALAC).

### Patients

The clinical descriptions are shown in Table 1. Patients 3 and 6 were of Hispanic ethnicity and the remainder were Caucasians. Patients 2–8 had sporadic ACDMPV and patient 1 represents a familial case with neither point mutation in *FOXF1* nor deletion in the 16q24.1 region identified. Patients 3 & 4 and 2, 5, & 6 were reported by Stankiewicz et al (2009) (pts D4 and M4) and by Sen et al (2013) (pts 15, 1, and 2), respectively. Patients 2 and 5 had missense mutations, patient 4 had a no stop mutation, patient 6 had a nonsense mutation and patient 3 had an approximately 1 Mb deletion involving *FOXF1* as well its upstream regulatory region. Patients 2–6 had associated anomalies of the gastrointestinal system. Butterfly vertebrae were identified in the patients 3 and 6. In patient 1, autopsy was limited to heart and lung. No clinical or molecular information about patients 7 and 8 was available.

**Table 1:** ACDMPV patient sample information.

Patient	Gender	Ethnicity	Inheritance	Life span (days)	*FOXF1* molecular defect	Associated anomalies
P1	M	Caucasian	Familial	48	None	Limited autopsy
P2	M	Caucasian	Sporadic	7	Missense mutation (c.286G>A;p.V96M)	Malrotation of the intestine, atrial septal defect, thick distended bladder with muscular hypertrophy, mild bilateral hydroureter and hydronephrosis
P3	F	Hispanic	Sporadic	21	Deletion (chr16: 84,402,571–85,435,712) (hg18)	Imperforate anus, suspected malrotation of the intestine, bicornuate uterus, multiple butterfly vertebrae
P4	M	Caucasian	Sporadic	46	No stop mutation (c.1138T>C;*380Rext*73)	Malrotation of the colon, Meckel's diverticulum
P5	M	Caucasian	Sporadic	28	Missense mutation (c.145C>T;p.P49S)	Incomplete rotation of bowel, right colon unfixed
P6	F	Hispanic	Sporadic	18	Nonsense mutation (c.89C>A;p.S30*	Malrotation of the intestine, imperforate anus, butterfly vertebrae, ventricular septal defect

No clinical or genetic information is available for patients 7 and 8.

### Animals

*Foxf1* ^+/−^ mice with one *Foxf1* allele disrupted by deletion of the forkhead binding domain were generated (Figure S1) and maintained on a mixed 129-C57BL6/J background. The mid-day of vaginal plug identification was considered as embryonic day 0.5 and the mid-day when pups were born was considered as postnatal day (P) 0.5.

### RNA isolation

Total RNA was isolated from approximately 10 mg frozen lung samples from patients and normal control humans using TRIzol (Invitrogen). Controls C1-C4 are age-matched lung samples from individuals that had no lung problems and died of other reasons. Control samples C1-C3 are from females and control sample C4 is a male sample. Control sample C5 is a commercially available human total lung RNA (Life Technologies Cat. No# AM7968). The tissue was homogenized in microfuge tubes with a hand held homogenizer in the presence of 200 μl of TRIzol. The aqueous phase was collected after spinning at 10,000 rpm for 5 min. This step was repeated and the pooled aqueous phase was loaded onto an RNeasy column (Qiagen). RNA was eluted from the columns following the manufacturer's instruction. The RNA was quantified using Nanodrop and its quality was assessed on a BioRad Bioanalyzer. In spite of the differences in their process of collection and preservation, the RNA isolated from the lung tissues of patients with ACDMPV were of good quality.

Mouse RNA was extracted from frozen whole lungs of P0.5 *Foxf1* ^+/−^ mice and their wildtype littermate controls. Whole lungs were homogenized using TissueLyser LT (Qiagen) in QIAzol lysis reagent (Qiagen) according to manufacturer's instructions. Total RNA was isolated from lung homogenates using the miRNeasy mini kit (Qiagen). RNAs were tested for quality on the Agilent 2100 bioanalyzer and quantified on the Nanodrop Spectrophotometer by the Genomic and RNA Profiling Core (GARP) facility at BCM.

### Microarray analysis

500 ng of total human RNA was converted into cDNA and then cRNA following the manufacturer's instructions. The cRNA was biotinylated and hybridized to the Human Ref8.V3 microarray (Illumina) contains probes representing ∼22,000 curated genes and ESTs conjugated with streptavidin-Cy3. Quality standards for hybridization, labeling, staining, background signal, and basal level of housekeeping gene expression for each chip were verified. The scanned images of the chip were analyzed using GenomeStudio software (Illumina). Gene expression analyses of RNA from patients' lungs (n = 8) was compared to those from the control lungs (n = 5).

Transcriptomes of six *Foxf1* ^+/−^ and six wildtype littermate samples were assessed in two separate microarray experiments using MouseWG-6 v2.0 Expression BeadChip kit (Illumina) containing probes for over 45,200 transcripts. 500 ng of total RNA was labeled using Illumina TotalPrep RNA Amplification Kit (Ambion) and hybridized as per manufacturer's instructions. The array data were analyzed using the lumi bioconductor package [21], normalized by robust spline normalization (RST), and transformed using variance stabilization transformation (VST) [22]. ComBAT batch correction [23] was applied to correct for differences in expression values between the two separate microarray experiments. Two-sample t-test was applied to determine differentially expressed genes between *Foxf1* ^+/−^ and wild type lung groups. Differential expression *p*-values were adjusted for false discovery rates (FDR). Fold changes were calculated using reverse VST.

Database for Annotation, Visualization, and Integrated Discovery (DAVID), Ingenuity pathway analysis (IPA), and Gene set enrichment analysis (GSEA) softwares were used for gene ontology, pathway, and network analyses.

The ACDMPV and *Foxf1* ^+/−^ lung microarray data sets can be accessed through the NCBI Gene Expression Omnibus (GEO) (http://www.ncbi.nih.gov/geo/) under accession numbers GSE54780 and GSE54128, respectively.

### qRT-PCR

Confirmation of the differential gene expression was done by qRT-PCR using an ABI 7900HT Fast real time PCR System. Universal probe library and Fast start probe master mix from Roche Applied Science was used following manufacturer's instructions.

For the mouse microarray analysis validation, RNA was reverse transcribed to cDNA using the SuperScript III First-Strand Synthesis System (Life Technologies) and amplified using the Power SYBR Green PCR Master Mix (Life Technologies). Primers used for qRT-PCR validation of the mouse microarray are listed in table S5.

### Immunohistochemistry

Antibody against COL1A1 was obtained from Sigma-Aldrich. The respective dilutions of antibodies and other experimental conditions used were following manufacturer's instructions.

## Results

### ACDMPV lung gene expression analysis

**Figure 1:**
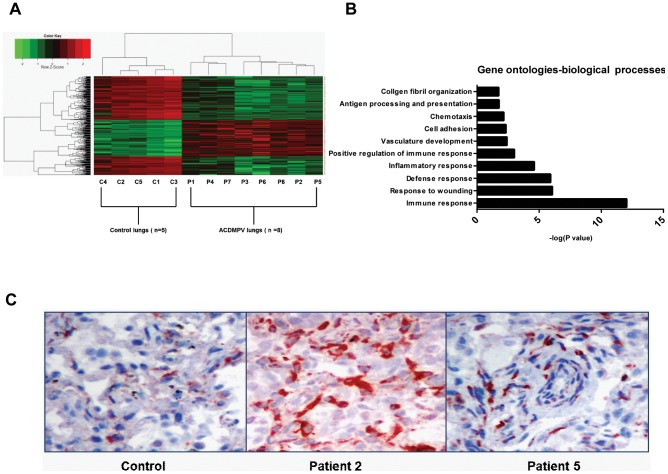
ACDMPV lung microarray analysis: heat map, clustering, DAVID analysis, and IHC validation. (A) Heat map for genes differentially expressed (adjusted p-value <0.05, fold change approximately >/ =  2 or </ =  − 2) between ACDMPV lungs (n  = 8) and control lungs (n = 5); Decreased gene expression is shown in green and increased gene expression is shown in red. (B) Top significant gene ontology (biological processes) terms associated with deregulated genes identified by DAVID analysis. (C) IHC validation for COL1A1; Representative slides from two patients' and one control sample at 20× magnification. A commercial control lung sample C5 clustered with the age-matched control lung samples C1-4. A lung sample from a patient who died from a different congenital diffuse lung developmental disorder was also included in the analysis. The deregulated genes in this sample clustered differently than those in ACDMPV lung samples (data not shown), confirming the validity of our approach.

Statistical analysis revealed 337 genes deregulated in ACDMPV lungs compared to control lungs (adjusted p-value <0.05, fold-change approximately >/ =  2 or </ =  −2), of which 205 (60.83%) were down-regulated and 132 (39.17%) were up-regulated (Figure 1A, Table S1). DAVID analysis revealed gene ontologies (GO) related to inflammation and immune responses, vasculature development, cell adhesion and chemotaxis associated with the deregulated genes (Figure 1B). The microarray data were validated using qRT-PCR for eight differentially expressed genes: *IL8*, *SPP1*, *IGFBP3*, *COL1A1*, *SOD2*, *GLI2*, *ANGPTL4,* and *HES4* (data not shown). Further, differences in protein quantity were assessed by immunohistochemistry (IHC) using antibodies against a significantly differentially expressed (*COL1A1*) product (Figure 1C). The amount of COL1A1 was significantly higher in the patient samples as compared to the control lung.

**Figure 2:**
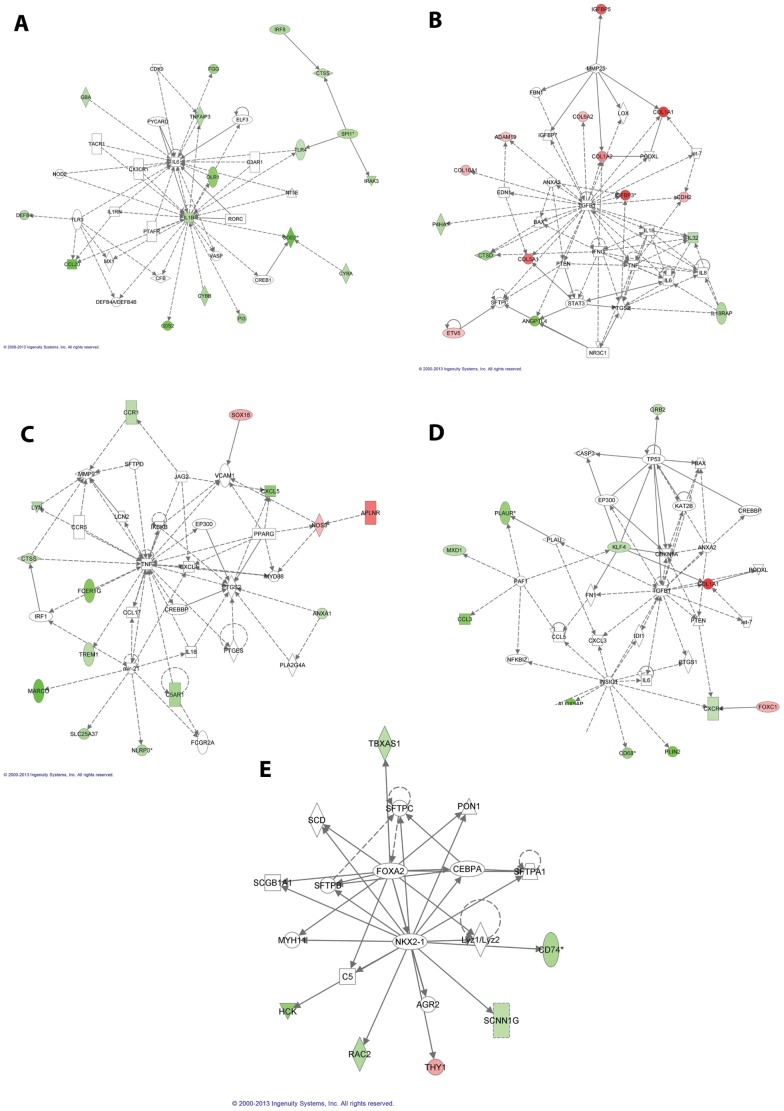
Networks involving deregulated genes in ACDMPV lungs identified using IPA; down-regulated genes are shown in green and up-regulated genes are shown in red. Deregulated genes involved in (A) Cellular movement, immune cell trafficking, inflammatory response; (B) Cellular development, cellular growth and proliferation, tumor morphology; (C) Cellular development, hematological system development and function, immune cell trafficking; (D) Cardiovascular disease, inflammatory response and cellular movement; (E) Carbohydrate metabolism, lipid metabolism and molecular transport.

GSEA and DAVID analyses showed enrichment of pathways related to signaling in the immune system, hemostasis, cell surface interactions at the vascular wall, and the lysosome (Table S2). Further analyses by GSEA allowed determining gene sets associated with chemical and genetic perturbations enriched in the 337 genes deregulated in the ACDMPV lungs (Table S3). Genes up-regulated in vascular smooth muscle cells by MAPK8, and genes up-regulated in the Kras2LA lung cancer mouse model, are enriched in the list of down-regulated genes. Genes up-regulated by E-cadherin, and genes down-regulated in the Kras2LA lung cancer mouse model are enriched in the list of up-regulated genes. IPA pathway analysis was used to build networks and demonstrate interactions among the deregulated genes (Figure 2). Top networks involve cellular movement, cellular proliferation, inflammatory response, cardiovascular disease, hematological system development, and carbohydrate and lipid metabolism.

### *Foxf1* ^+/−^ mouse lung gene expression analysis

**Figure 3:**
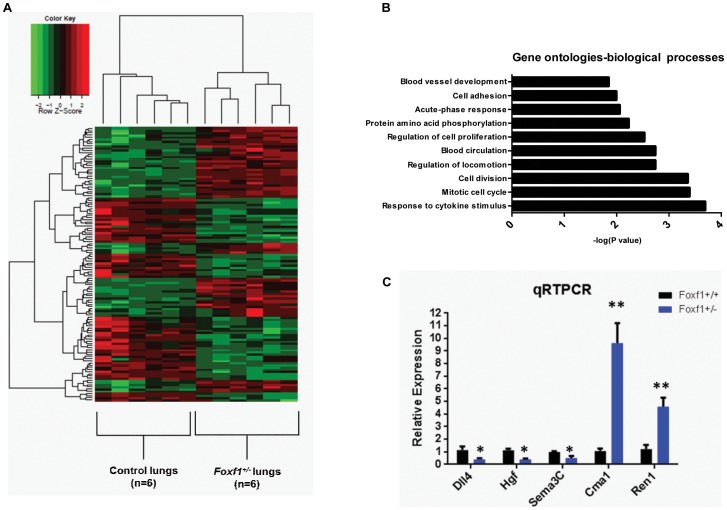
*Foxf1* ^+/−^ mouse lung microarray analysis: heat map, clustering, DAVID analysis, and qRT-PCR validation. (A) Heat map for genes differentially expressed (fdr <0.05, fold change >/ =  2 or </ =  − 2) between *Foxf1* ^+/−^ lungs (n = 6) and littermate control lungs (n = 6); Decreased gene expression is shown in green and increased gene expression is shown in red; *Foxf1* ^+/−^ lung samples and wildtype lung samples cluster as two separate groups. (B) Top significant gene ontology (biological processes) terms associated with deregulated genes from DAVID analysis. (C) qRT-PCR validation for down-regulated genes (*Sema3C, Dll4,* and *Hgf*) and up-regulated genes *(Cma1* and *Ren1*); n = 6 for each group; * =  p value <0.05; ** = p value <0.001.

Statistical analysis showed 789 deregulated genes in *Foxf1* ^+/−^ lungs compared to control lungs (FDR< 0.05, fold-change >/ =  1.5 and </ =  −1.5) (Figure 3A). 394 genes of them (49.93%) were down-regulated and 395 (50.06%) were up-regulated (Table S4). GSEA and DAVID analyses revealed GO terms related to response to cytokine stimulus, cell division, blood circulation, regulation of cell proliferation and cell adhesion associated with deregulated genes (Figure 3B).

**Figure 4:**
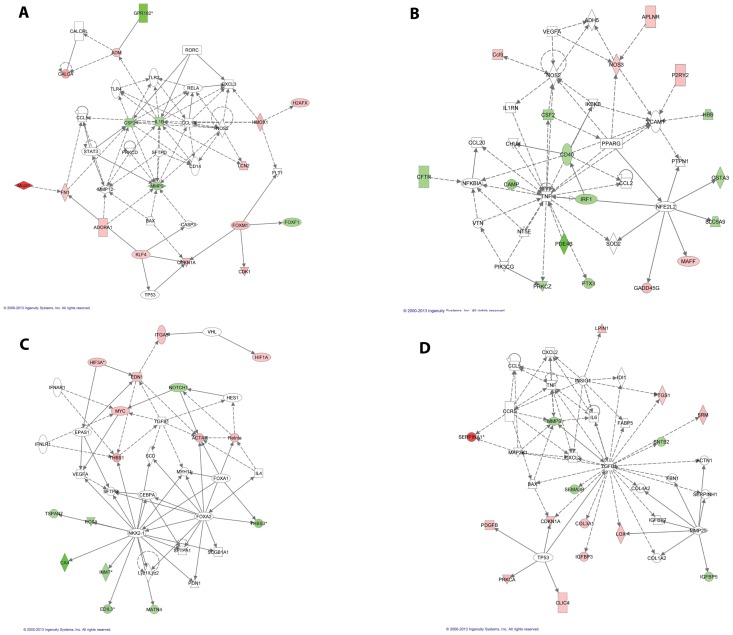
Networks involving deregulated genes in *Foxf1^+/−^* lungs identified using IPA. Deregulated genes involved in (A) Inflammatory response, cellular movement, cardiovascular disease; (B) Cell-to-cell signaling and interaction, cellular movement, hematological system development and function; (C) Embryonic development, organ development; (D) Cardiovascular system development and function, organ development, cellular movement; down-regulated genes are shown in green and up-regulated gens are shown in red.

The microarray data were confirmed using qRT-PCR for five differentially expressed genes: *Dll4, Hgf, Sema3C, Cma1,* and *Ren1* (Figure 3C). Canonical pathways affected include genes involved in focal adhesion, homeostasis, p53 signaling, as well as in Vegf, Endothelin, and Notch signaling (Table S6). Additional GSEA analysis identified genes down-regulated by E-cadherin and genes up-regulated in vascular smooth muscle cells by MAPK8 to be enriched in the list of down-regulated genes. Genes regulated by hypoxia were found to be enriched in the list of up-regulated genes (Table S7). Network analysis by IPA identified interactions amongst genes involved in cellular movement, inflammatory response, and hematological and cardiovascular system development (Figure 4).

### Gene expression alterations common to both ACDMPV and *Foxf1* ^+/−^ lung transcriptomes

**Figure 5:**
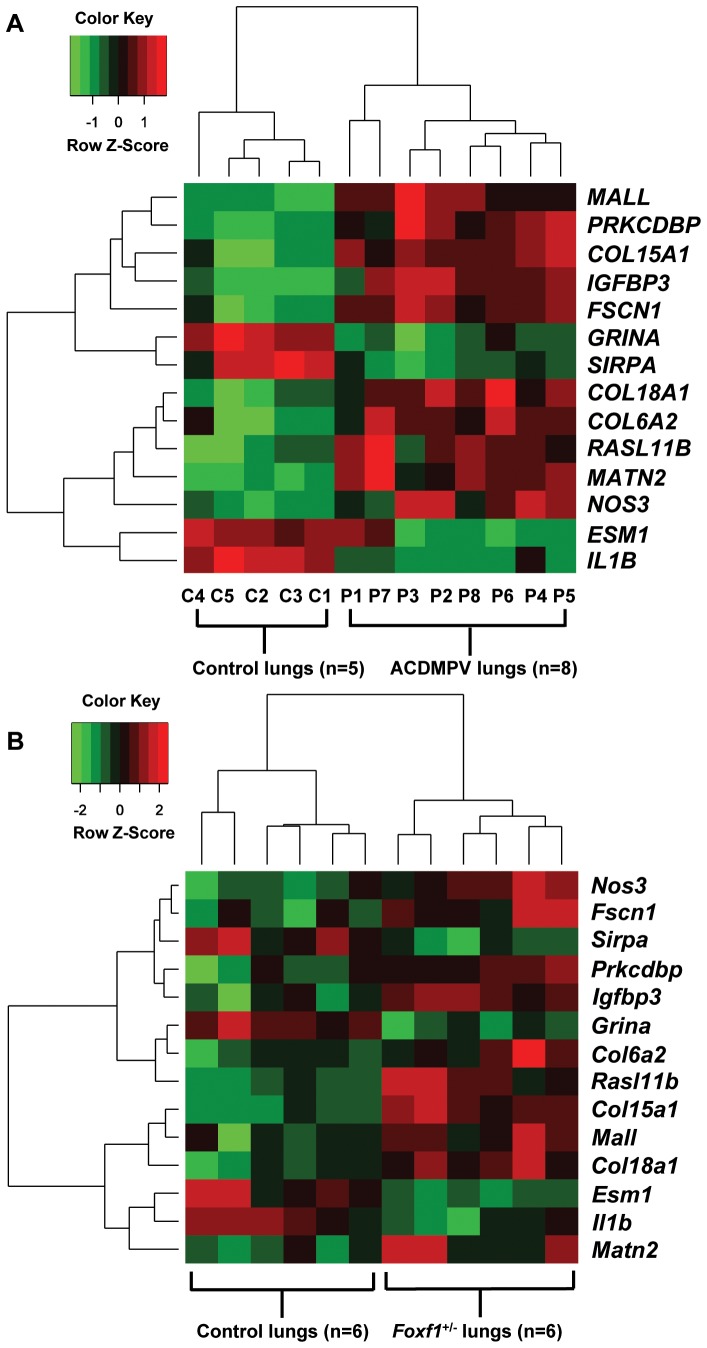
Deregulated genes common to both ACDMPV lungs and P0.5 *Foxf1^+/−^* lungs. **(A)** Heat map comparing expression of 14 overlapping genes in ACDMPV lung samples and control lung samples; (B) Heat map comparing expression of 14 overlapping genes in *Foxf1* ^+/−^ lung samples and control lung samples.

To identify genes regulated by both *FOXF1* and *Foxf1* in human and mouse lungs, respectively, we compared genes with similarly altered expression in both ACDMPV patients and P0.5 *Foxf1* ^+/−^ pups and identified fourteen intersecting genes (Figure 5; Table S8). Ten genes: *COL15A1, COL18A1, COL6A2, FSCN1, IGFBP3, MALL, MATN2, NOS3, PRKCDBP,* and *RASL11B* upregulated in ACDMPV patients were also upregulated in *Foxf1* ^+/−^ pups. Four genes: *ESM1, GRINA, IL1B,* and *SIRPA* were downregulated in ACDMPV patients and also *Foxf1* ^+/−^ pups. However, five genes *ORM1, FGG, KLF4, IGFBP5*, and *GAMT* were found to be differentially altered in the human vs mouse microarray datasets.

## Discussion

To date, we have recruited greater than 100 families with children affected with ACDMPV. This is the largest collection of ACDMPV samples worldwide. To identify transcriptional changes in the neonate ACDMPV lungs, we compared the whole genome expression in the lungs of eight patients with ACDMPV with transcriptional changes in P0.5 *Foxf1^+/−^* mouse lungs, and appropriate control lungs. This approach enabled us to identify gene ontologies and pathways associated with ACDMPV and gain a better understanding of the disease.

The *Foxf1^+/−^* mouse lungs showed down-regulation of Notch signaling genes *Dll4* and *Notch1* and slightly lower but significant (fold change: −1.46) down-regulation of the Notch target gene *Hey1* (data not shown). Notch signaling has been reported to be affected in *Foxf1^+/−^* lungs [24]. Genes involved in pulmonary vasculature development, *Sema3c* and lung branching morphogenesis, *Hgf* and *Lama1*, were also down-regulated. *Sema3c,* a semaphorin gene expressed in the lung endothelium, interacts with neuropillin-1 during vasculature development; loss of semaphorin-neuropilin-1 signaling showed lung pathology reminiscent of ACDMPV [25]. *Sema3c* was down-regulated in our study and *Foxf1* was down-regulated in lungs of *Nrp1^Sema-^* mice, suggesting a possible interaction between *Foxf1* and *Sema3C* in murine lung pathology. These transcriptional changes potentially lead to underdeveloped lungs causing respiratory distress at birth.

Transcriptional changes found in *Foxf1* ^+/−^ lungs, potentially resulting from the respiratory distress, include deregulation of pathways associated with hypoxia-induced vascular remodeling. There is increased expression of α-smooth muscle actin *Acta2*, indicating greater muscularization of pulmonary vessels and possible pulmonary hypertension. Greater expression of *Acta2* could also be due to endothelium-to-mesenchyme transition that is found to accompany vascular remodeling in pulmonary hypertension [26]. Additionally, members of the Endothelin-1 signaling pathway were deregulated. Endothelin-1 (ET-1) is a potent vasoconstrictor regulating vascular tone and is implicated in pulmonary hypertension. *Edn1* over-expression in mice leads to development of systemic hypertension with altered vascular reactivity [27].

Fibronectin and several members of the collagen gene family are also found to be up-regulated, likely leading to increased production of extracellular matrix (ECM) proteins. Accumulation of ECM proteins such as collagen, elastin, and fibronectin leads to thickening of pulmonary blood vessels, likely contributing to pulmonary hypertension. Interestingly, endothelin induced collagen remodeling has been reported in a mouse model of pulmonary hypertension [28], indicating that the increased endothelin-1 signaling in *Foxf1* ^+/−^ lungs may be causing an increase in collagen production. Integrins involved in cell surface interactions at the vascular wall were also found to be down-regulated. Similar transcriptional changes have been observed to be associated with matrix and vasculature remodeling in TGF-α induced pulmonary fibrosis [29]. *Foxf1* was shown to play an essential role in mesenchymal migration by transcriptionally regulating integrin-β3 (Itgβ3) [30]. Integrin-ECM interactions also contribute to vascular remodeling. Altered integrin-ECM interactions have been documented in chronic hypoxia and monocrotaline-induced pulmonary hypertension [31].

Mast cell chymase and tryptase genes (*Mcpt4, Mcpt6, Cma1,* and *Cpa3*) are highly up-regulated i n *Foxf1^+/−^* lungs, showing evidence of pulmonary mastocytosis. Pulmonary mastocytosis and enhanced lung inflammation in *Foxf1^+/−^* mice after chemically induced or allergen-mediated lung injury was reported [32]. These inflammatory molecules could contribute to the vascular remodeling through their effects on vascular cell wall permeability, matrix protein production, and by inducing recruitment of mesenchymal precursor cells [33]. Similar accumulation of mast cell mediators was found in lungs of *Fgfr3/4* mutant mice that exhibit Bronchopulmonary Dysplasia (BPD) like pathology and in lungs of children with BPD [34], suggesting that pulmonary mastocytosis could be a common feature found in diffuse lung developmental conditions. *Ren1* which along with *Cma1* and *Cpa3* is a component of the blood pressure regulating renin angiotensin system is also up-regulated.

Other transcriptional changes include up-regulation of *Mki67*, *Ccnd1,* and *Foxm1*, showing an increase in proliferation in the lung. *Foxf1* has been reported to be down-regulated in mice with endothelial cell-specific deletion of *Foxm1* [35], indicating a reciprocal feedback loop between *Foxf1* and *Foxm1.* Finally, up-regulation of *Hif3α* and *Nos3* is also observed, suggesting a compensatory mechanism to counteract the effects of hypoxia-induced pulmonary hypertension.

Microarray analysis of ACDMPV human lung transcriptomes primarily identified deregulation of genes involved in inflammation and cell surface interactions at the vascular wall. Members of the collagen gene family are highly up-regulated similar to the up-regulation seen in *Foxf1* ^+/−^ lungs, potentially leading to increased ECM collagen production, causing thickening of pulmonary blood vessels, and resulting in pulmonary hypertension, a typical feature found in neonates with ACDMPV.

IHC for COL1A1 confirmed the increased protein levels of COL1A1 in ACDMPV patient lungs (Figure 1C). Structural changes in ACDMPV lungs that could be attributed to hypoxia-induced vascular remodeling include medial thickening of smooth muscles in pulmonary arteries. Transcriptional changes which could be described as compensatory mechanisms to counteract the pulmonary hypertension include down-regulation of genes involved in TREM1 signaling (*TREM1, TLR4, IL1B,* and *ITGAX*) and up-regulation of *NOS3*. Pulmonary inflammatory responses play an important role in hypoxia-induced vascular remodeling [33] and down-regulation of TREM1 signaling could be a compensatory response to attenuate pulmonary inflammatory responses. *Tlr-4* deficient mice were shown to be resistant to chronic hypoxia-induced pulmonary hypertension by attenuating the pulmonary vascular inflammatory response to hypoxia [36]. Up-regulation of *NOS3* also could be a compensatory response to counteract the hypertension.

Other affected pathways include signaling related to the lysosome function, carbohydrate metabolism, cell cycle, and axon guidance which are also deregulated in *Foxf1* ^+/−^ lungs. Interestingly, we found targets of E-cadherin enriched in the deregulated genes. Recently, E-cadherin has been shown to be a target of FOXF1, which in turn has been shown to be a target of p53, regulating cancer cell migration and invasiveness [37]. Additionally, *FOXF1* expression has been shown to be changed in numerous human malignancies [38]–[45].

Comparison of the deregulated genes in both experiments enabled identification of 14 genes, *COL15A1, COL18A1, COL6A2, ESM1, FSCN1, GRINA, IGFBP3, IL1B, MALL, NOS3, RASL11B, MATN2, PRKCDBP,* and *SIRPA,* similarly altered in both ACDMPV and *Foxf1* heterozygous lung transcriptomes. *ESM1* is mainly expressed in endothelial cells and its expression is upregulated by cytokines like TNFα and IL-1β suggesting that it plays a role in endothelium-dependent pathological disorders [46]. *ESM1* as well as *IL1B* are downregulated in both the microarray datasets. *ESM1* has also been computationally predicted to have a FOX: ETS binding motif in its vicinity suggesting that it could be a direct target of *FOXF1* in endothelial cells [47]. Another common downregulated gene in both datasets, *SIRPA* has been shown to interact with surfactant proteins A & D in the absence of inflammation to suppress alveolar macrophage phagocytosis [48]. Whereas there is greater variation for *Sirpa* in the mouse data, the average fold change difference for this gene correlates with the human data.

In aggregate, we present the results of genome-wide analyses of transcriptional changes in lungs from patients with ACDMPV due to point mutations or genomic deletions in *FOXF1* and in mice heterozygous for *Foxf1* loss. The transcriptional changes should help better define the molecular mechanisms involved in the pathogenesis of ACDMPV and may also be a valuable source for future research on lung vasculature development and pulmonary disease conditions such as pulmonary hypertension, bronchopulmonary dysplasia, and lung cancer.

## Supporting Information

Figure S1 **Generation of the** ***Foxf1*** **^+/−^ mouse line.** Single allele deletion of the *Foxf1* gene was achieved by breeding of *Foxf1*-floxed heterozygous females with EIIa-Cre male mice. *Foxf1*-floxed allele possesses two LoxP sites (white arrowhead) and one Frt site that surround exon 1, encoding the Forkhead DNA-binding domain of the *Foxf1* protein. Cre-mediated recombination occurs in a wide range of tissues, including the germ cells that transmit *Foxf1*-null allele to offspring. Exon 1 and part of the promoter of *Foxf1* are deleted. The GRCm38/mm10 coordinates of the deleted region are chr8∶121,083,499-121,085,436 (1938 bp). *Fendrr* is a long non coding RNA gene located upstream to *Foxf1*, and negatively regulates it. Primer sequences used to genotype mice with heterozygous loss of *Foxf1* are: F: 5′-TTCAGATCTGAGAGTGGCAGCTTC-3′; R1∶5′-GCTTTGTCTCCAAGCGCTGC-3′; and R2∶5′GAAGGAACCCAGATGTTCCCTG-3′.
(TIF)

Table S1 **ACDMPV lung microarray analysis: List of deregulated genes (adjusted p value <0.05, fold change> / =  2 or </ =  - 2).**
(XLSX)

Table S2 **Pathways enriched in ACDMPV lung transcriptomes: GSEA and DAVID analyses (p-value <0.05).**
(XLSX)

Table S3 **Chemical and genetic perturbation gene sets enriched in ACDMPV lungs: GSEA analysis (fdr <0.05).**
(XLSX)

Table S4 ***Foxf1*** **^+/−^ mouse lung microarray analysis: List of deregulated genes (fdr <0.05, fold change > / =  1.5 or </ =  − 1.5).**
(XLSX)

Table S5 **List of primer sequences used for qRT-PCR validation using SYBR.**
(XLSX)

Table S6 **Pathways enriched in** ***Foxf1^+/−^*** **lung transcriptomes: GSEA and DAVID analyses (p-value <0.05).**
(XLSX)

Table S7 **Chemical and genetic perturbation gene sets enriched in** ***Foxf1^+/−^*** **lungs: GSEA analysis (fdr <0.05).**
(XLSX)

Table S8 **GO terms associated with genes similarly altered in both ACDMPV lungs and** ***Foxf1*** **^+/−^ lungs.**
(XLSX)
