# Supplementary figures and images for: Comparative Analyses of Lung Transcriptomes in Patients with Alveolar Capillary Dysplasia with Misalignment of Pulmonary Veins and in Foxf1 Heterozygous Knockout Mice
Source: PLoS One. 2014 Apr 10;9(4):e94390. doi: 10.1371/journal.pone.0094390 (PMC3983164; doi:10.1371/journal.pone.0094390)

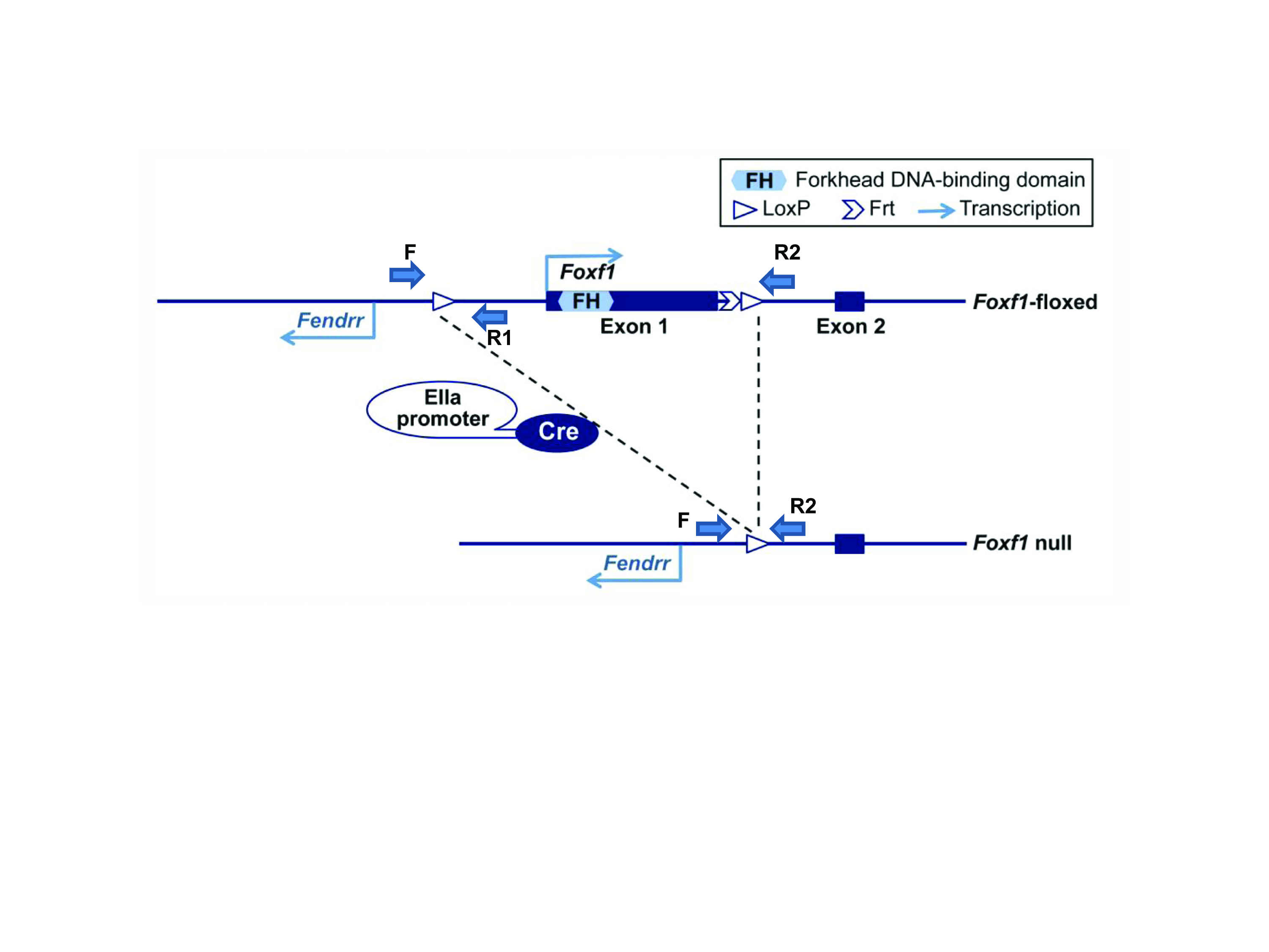

Supplement: Figure S1 — Generation of the Foxf1 +/− mouse line. Single allele deletion of the Foxf1 gene was achieved by breeding of Foxf1-floxed heterozygous females with EIIa-Cre male mice. Foxf1-floxed allele possesses two LoxP sites (white arrowhead) and one Frt site that surround exon 1, encoding the Forkhead DNA-binding domain of the Foxf1 protein. Cre-mediated recombination occurs in a wide range of tissues, including the germ cells that transmit Foxf1-null allele to offspring. Exon 1 and part of the promoter of Foxf1 are deleted. The GRCm38/mm10 coordinates of the deleted region are chr8∶121,083,499-121,085,436 (1938 bp). Fendrr is a long non coding RNA gene located upstream to Foxf1, and negatively regulates it. Primer sequences used to genotype mice with heterozygous loss of Foxf1 are: F: 5′-TTCAGATCTGAGAGTGGCAGCTTC-3′; R1∶5′-GCTTTGTCTCCAAGCGCTGC-3′; and R2∶5′GAAGGAACCCAGATGTTCCCTG-3′. (TIF) [file pone.0094390.s001.tif]
